# Assessment of Clinical Outcomes Among Children and Adolescents Hospitalized With COVID-19 in 6 Sub-Saharan African Countries

**DOI:** 10.1001/jamapediatrics.2021.6436

**Published:** 2022-01-19

**Authors:** Jean B. Nachega, Nadia A. Sam-Agudu, Rhoderick N. Machekano, Helena Rabie, Marieke M. van der Zalm, Andrew Redfern, Angela Dramowski, Natasha O’Connell, Michel Tshiasuma Pipo, Marc B. Tshilanda, Liliane Nsuli Byamungu, Refiloe Masekela, Prakash Mohan Jeena, Ashendri Pillay, Onesmus W. Gachuno, John Kinuthia, Daniel Katuashi Ishoso, Emmanuella Amoako, Elizabeth Agyare, Evans K. Agbeno, Charles Martyn-Dickens, Justice Sylverken, Anthony Enimil, Aishatu Mohammed Jibril, Asara M. Abdullahi, Oma Amadi, Umar Mohammed Umar, Lovemore Nyasha Sigwadhi, Michel P. Hermans, John Otshudiema Otokoye, Placide Mbala-Kingebeni, Jean-Jacques Muyembe-Tamfum, Alimuddin Zumla, Nelson K. Sewankambo, Hellen Tukamuhebwa Aanyu, Philippa Musoke, Fatima Suleman, Prisca Adejumo, Emilia V. Noormahomed, Richard J. Deckelbaum, Mary Glenn Fowler, Léon Tshilolo, Gerald Smith, Edward J. Mills, Lawal W. Umar, Mark J. Siedner, Mariana Kruger, Philip J. Rosenthal, John W. Mellors, Lynne M. Mofenson

**Affiliations:** 1 Department of Epidemiology, Infectious Diseases and Microbiology, Center for Global Health, University of Pittsburgh, Pittsburgh, Pennsylvania; 2 Department of Medicine, Stellenbosch University Faculty of Medicine and Health Sciences, Cape Town, South Africa; 3 Department of Epidemiology, Johns Hopkins Bloomberg School of Public Health, Baltimore, Maryland; 4 Department of International Health, Johns Hopkins Bloomberg School of Public Health, Baltimore, Maryland; 5 International Research Center of Excellence, Institute of Human Virology Nigeria, Abuja, Nigeria; 6 Institute of Human Virology, University of Maryland School of Medicine, Baltimore; 7 Department of Pediatrics, University of Maryland School of Medicine, Baltimore; 8 Department of Pediatrics and Child Health, University of Cape Coast School of Medical Sciences, Cape Coast, Ghana; 9 Division of Epidemiology and Biostatics, Department of Global Health, Stellenbosch University Faculty of Medicine and Health Sciences, Cape Town, South Africa; 10 Department of Pediatrics and Child Health, Stellenbosch University, Cape Town, South Africa; 11 Desmond Tutu TB Centre, Department of Pediatrics and Child Health, Stellenbosch University, Cape Town, South Africa; 12 Department of Public Health, Centre Interdisciplinaire de Recherche en Ethnopharmacologie, Faculty of Medicine, Université Notre-Dame du Kasayi, Kananga, Democratic Republic of the Congo; 13 Unit of Sickle Cell Disease and Clinical Research, Monkole Hospital Center, Kinshasa, Democratic Republic of the Congo; 14 Department of Pediatrics and Child Health, School of Clinical Medicine, College of Health Sciences, University of KwaZulu Natal, Durban, South Africa; 15 Department of Obstetrics and Gynecology, University of Nairobi, Nairobi, Kenya; 16 Department of Research and Programs, Kenyatta National Hospital, Nairobi, Kenya; 17 Community Health Department, Kinshasa School of Public Health, University of Kinshasa, Kinshasa, Democratic Republic of the Congo; 18 Department of Pediatrics, Cape Coast Teaching Hospital, Cape Coast, Ghana; 19 Department of Microbiology, School of Medical Sciences, University of Cape Coast and Cape Coast Teaching Hospital, Cape Coast, Ghana; 20 Department of Obstetrics and Gynecology, School of Medical Sciences, University of Cape Coast and Cape Coast Teaching Hospital, Cape Coast, Ghana; 21 Pediatrics Infectious Diseases Unit, Komfo Anokye Teaching Hospital, Kumasi, Ghana; 22 Department of Child Health, School of Medicine and Dentistry, Kwame Nkrumah University of Science and Technology, Kumasi, Ghana; 23 Department of Pediatrics, College of Medical Sciences, Ahmadu Bello University, Zaria, Nigeria; 24 Department of Internal Medicine, College of Medical Sciences, Ahmadu Bello University, Zaria, Nigeria; 25 Department of Pediatrics, Asokoro District Hospital, Abuja, Nigeria; 26 Department of Internal Medicine, Asokoro District Hospital, Abuja, Nigeria; 27 Department of Endocrinology and Nutrition, Cliniques Universitaires St-Luc, Brussels, Belgium; 28 Health Emergencies Program, COVID-19 Response, World Health Organization, Kinshasa, Democratic Republic of the Congo; 29 National Institute of Biomedical Research, Department of Medical Microbiology and Virology, Faculty of Medicine, University of Kinshasa, Kinshasa, Democratic Republic of the Congo; 30 Division of Infection and Immunity, Centre for Clinical Microbiology, University College London, London, United Kingdom; 31 National Institute for Health Research Biomedical Research Centre, University College London Hospitals National Health Services Foundation Trust, London, United Kingdom; 32 School of Medicine, College of Health Sciences, Makerere University, Kampala, Uganda; 33 Department of Pediatrics, Mulago Hospital, Kampala, Uganda; 34 Department of Pediatrics and Child Health, College of Health Sciences, Makerere University, Kampala, Uganda; 35 Discipline of Pharmaceutical Sciences, University of KwaZulu Natal, Durban, South Africa; 36 Department of Nursing, University of Ibadan, Ibadan, Nigeria; 37 Faculty of Medicine, Eduardo Mondlane University, Maputo, Mozambique; 38 Department of Pediatrics, Institute of Human Nutrition, Columbia University Irving Medical Center, New York, New York; 39 Department of Pathology, Johns Hopkins University School of Medicine, Baltimore, Maryland; 40 Department of Pediatrics, Official University of Mbuji-Mayi, Kinshasa, Democratic Republic of the Congo; 41 Le Centre de Formation et d'Appui Sanitaire, Centre Hospitalier Monkole, Kinshasa, Democratic Republic of the Congo; 42 Department of Real World and Advanced Analytics, Cytel, Vancouver, British Columbia, Canada; 43 Department of Health Research Methods, Evidence and Impact, Faculty of Health Sciences, McMaster University, Hamilton, Ontario, Canada; 44 Department of Medicine, Division of Infectious Diseases, Harvard Medical School, Massachusetts General Hospital, Boston; 45 Department of Medicine, School of Medicine, Mbarara University of Science and Technology, Mbarara, Uganda; 46 Department of Medicine, Division of Infectious Diseases, University of California, San Francisco, San Francisco; 47 Department of Medicine, Division of Infectious Diseases, University of Pittsburgh School of Medicine, Pittsburgh, Pennsylvania; 48 Elizabeth Glaser Pediatric AIDS Foundation, Washington, District of Columbia

## Abstract

**Importance:**

Little is known about COVID-19 outcomes among children and adolescents in sub-Saharan Africa, where preexisting comorbidities are prevalent.

**Objective:**

To assess the clinical outcomes and factors associated with outcomes among children and adolescents hospitalized with COVID-19 in 6 countries in sub-Saharan Africa.

**Design, Setting, and Participants:**

This cohort study was a retrospective record review of data from 25 hospitals in the Democratic Republic of the Congo, Ghana, Kenya, Nigeria, South Africa, and Uganda from March 1 to December 31, 2020, and included 469 hospitalized patients aged 0 to 19 years with SARS-CoV-2 infection.

**Exposures:**

Age, sex, preexisting comorbidities, and region of residence.

**Main Outcomes and Measures:**

An ordinal primary outcome scale was used comprising 5 categories: (1) hospitalization without oxygen supplementation, (2) hospitalization with oxygen supplementation, (3) ICU admission, (4) invasive mechanical ventilation, and (5) death. The secondary outcome was length of hospital stay.

**Results:**

Among 469 hospitalized children and adolescents, the median age was 5.9 years (IQR, 1.6-11.1 years); 245 patients (52.4%) were male, and 115 (24.5%) had comorbidities. A total of 39 patients (8.3%) were from central Africa, 172 (36.7%) from eastern Africa, 208 (44.3%) from southern Africa, and 50 (10.7%) from western Africa. Eighteen patients had suspected (n = 6) or confirmed (n = 12) multisystem inflammatory syndrome in children. Thirty-nine patients (8.3%) died, including 22 of 69 patients (31.9%) who required intensive care unit admission and 4 of 18 patients (22.2%) with suspected or confirmed multisystem inflammatory syndrome in children. Among 468 patients, 418 (89.3%) were discharged, and 16 (3.4%) remained hospitalized. The likelihood of outcomes with higher vs lower severity among children younger than 1 year expressed as adjusted odds ratio (aOR) was 4.89 (95% CI, 1.44-16.61) times higher than that of adolescents aged 15 to 19 years. The presence of hypertension (aOR, 5.91; 95% CI, 1.89-18.50), chronic lung disease (aOR, 2.97; 95% CI, 1.65-5.37), or a hematological disorder (aOR, 3.10; 95% CI, 1.04-9.24) was associated with severe outcomes. Age younger than 1 year (adjusted subdistribution hazard ratio [asHR], 0.48; 95% CI, 0.27-0.87), the presence of 1 comorbidity (asHR, 0.54; 95% CI, 0.40-0.72), and the presence of 2 or more comorbidities (asHR, 0.26; 95% CI, 0.18-0.38) were associated with reduced rates of hospital discharge.

**Conclusions and Relevance:**

In this cohort study of children and adolescents hospitalized with COVID-19 in sub-Saharan Africa, high rates of morbidity and mortality were observed among infants and patients with noncommunicable disease comorbidities, suggesting that COVID-19 vaccination and therapeutic interventions are needed for young populations in this region.

## Key Points

**Question:**

What are the clinical outcomes and associated factors among children and adolescents hospitalized with COVID-19 in sub-Saharan Africa?

**Findings:**

In this cohort study of 469 children and adolescents hospitalized with COVID-19 in 6 sub-Saharan African countries, morbidity and mortality were substantially higher than reported among those in non-African settings and were independently associated with age younger than 1 year and select noncommunicable disease comorbidities.

**Meaning:**

This study’s findings may have implications for clinical practice and health policy regarding pediatric COVID-19 in African countries; given their high risk of adverse outcomes, COVID-19 vaccination and therapeutic interventions are needed for African children and adolescents.

## Teaser

This cohort study assesses clinical outcomes and associated factors among children and adolescents hospitalized with COVID-19 in 6 sub-Saharan African countries.

## Introduction

After 2 years of the COVID-19 pandemic, several studies^1,2,3,4,5,6,7,8,9^ have reported that disease severity is substantially lower among children compared with adults. Of the more than 315 million cases and 5.5 million deaths reported to be associated with SARS-CoV-2 as of January 13, 2022, more than 29 million cases and 22 000 deaths are estimated among children and adolescents aged 0 to 19 years.^10^ Data primarily from China, Italy, the UK, the US, and several European countries have revealed that between 1% and 5% of all COVID-19 cases occur in children, with an overall mortality rate of 1% or lower in hospitalized children.^1,2,3,4,5,6,7,8,9^

The African continent has a young population; children younger than 18 years constitute almost 50% of people.^11^ Despite increasing knowledge about COVID-19 in children, data from African countries are limited.^12,13,14,15^ Most reports about COVID-19 among African children have been small single-center studies with scarce data on clinical presentation and outcomes.^14,16,17,18^ Sub-Saharan Africa has a high prevalence of both communicable (eg, HIV infection and tuberculosis) and noncommunicable (eg, asthma, cancer, diabetes, hypertension, and sickle cell anemia) diseases that also occur among children.^19,20^ Combined with the high prevalence of comorbidities, limited availability of intensive care may have substantial consequences for COVID-19 outcomes in sub-Saharan Africa.^20,21^ In the multicenter African COVID-19 Critical Care Outcomes Study,^22^ almost 50% of adults with COVID-19 died within 30 days of intensive care unit (ICU) admission, with up to 23 excess deaths per 100 patients compared with the global average. Limited critical care resources, organ dysfunction at admission, and select comorbidities accounted for this excess mortality. Both children and adults experience inadequate availability of and access to SARS-CoV-2 testing and high-quality intensive care in constrained sub-Saharan African settings.^15,22,23^ Access to hospital care is limited and varies within and across countries and regions.^24,25^ The burden of SARS-CoV-2 infection, including severe disease requiring hospitalization, is underestimated in sub-Saharan Africa^26,27,28^ and is potentially more underestimated among children, who are less likely to be evaluated for infection.^15^ To address this issue, the present study assessed clinical manifestations, outcomes, and factors associated with outcomes among children and adolescents hospitalized with COVID-19 in 6 countries in sub-Saharan Africa.

## Methods

This cohort study was a multicountry retrospective record review that pooled data from hospitalized children and adolescents aged 0 to 19 years with SARS-CoV-2 infection confirmed through reverse transcriptase polymerase chain reaction testing. Study review and approval, including waivers of informed consent and permission to use deidentified information from existing data sets or medical records, were obtained from institutional and/or national research ethics committees and/or regulatory bodies in participating countries (eTable 2 in Supplement 1). This study followed the Strengthening the Reporting of Observational Studies in Epidemiology (STROBE) reporting guideline for cohort studies.^29^

### Settings and Participants

The study included all children and adolescents with confirmed SARS-CoV-2 infection who were admitted to 25 health care facilities in the Democratic Republic of the Congo (7 facilities), Ghana (2 facilities), Kenya (1 facility), Nigeria (2 facilities), South Africa (10 facilities), and Uganda (3 facilities) between March 1 and December 31, 2020. These countries were selected based on regional representation (eastern, western, central, and southern Africa) to participate in the study. For between-country comparisons of outcomes, western and central African regions were combined to maximize available sample size and statistical power. Data on race and ethnicity were not collected because the racial profile across the 6 countries was more than 90% Black or African descent, and the ethnic diversity across the 6 countries was too broad (almost 750 ethnic groups) for meaningful categorization or analysis. Detailed information about participating health care facilities (including names, locations, urban vs rural settings, and public vs private status) is available in eTable 1 and eFigure 1 in Supplement 1.

### Variables

Using World Health Organization (WHO) pediatric COVID-19 case report forms,^30^ demographic and clinical data were extracted from national or institutional COVID-19 data sets and/or hospital records. Data collected included age, sex, preexisting comorbidities, WHO-defined COVID-19 severity stage at admission,^31^ and diagnosis of multisystem inflammatory syndrome in children (MIS-C) temporally associated with COVID-19.^30,32^ To accommodate partial or complete lack of laboratory and imaging data (eg, inflammatory markers and echocardiographic results) required for MIS-C diagnosis in our study settings, cases were characterized as suspected MIS-C when at least 2 required multisystem abnormalities that were clinically observable or measurable were documented in the medical records and/or databases from which study data were extracted. This requirement was in addition to fulfilling WHO criteria for the diagnosis of MIS-C that pertained to ruling out “other obvious microbial cause[s] of inflammation”^32^^(p1)^ plus confirmation of COVID-19 through a positive result on reverse transcriptase polymerase chain reaction testing.

### Outcomes

We selected an ordinal scale primary outcome with 5 ordered categories: (1) hospitalization without oxygen supplementation, (2) hospitalization with oxygen supplementation, (3) ICU admission, (4) invasive mechanical ventilation, and (5) death. This 5-scale primary outcome provided a measure of COVID-19 illness severity ranging from 1 (mild disease) to 5 (death). The secondary outcome was length of hospital stay.

### Statistical Analysis

Baseline demographic and clinical characteristics were summarized using frequencies and proportions; medians and IQRs were applied to categorical and continuous variables. For missing data on preexisting comorbidities, we performed multiple imputation using chained equations to generate 20 data sets. Most comorbidities had missingness less than 10%, with the exception of diabetes (23%), chronic lung disease (26%), cerebral palsy (28%), and cardiac disease (35%).

Multivariable proportional odds logistic regression analysis was used to identify factors associated with outcome severity among those with SARS-CoV-2 infection by including only factors that were considered clinically relevant and had a significance level of *P* < .15 in bivariable analyses. In our analyses, the proportional odds logistic regression model compared lower severity levels with higher severity levels (eg, category 1 vs categories 2-5, categories 1 and 2 vs categories 3-5, categories 1-3 vs categories 4 and 5, or categories 1-4 vs category 5). The proportional odds assumption was evaluated using χ^2^ and parallel line tests. Using robust SEs, the bivariable and multivariable proportional odds logistic models were fitted to account for potential within-cluster correlation of outcomes owing to shared processes and quality of care. Adjusted odds ratios (aORs) and associated 95% CIs were used to characterize the association between factors and disease severity.

We examined factors associated with the probability of hospital discharge over time using a competing-risk analysis of the Fine and Gray proportional subdistribution hazards model^33^ accounting for death. Factors with significance levels of *P* < .15 in bivariate models were included in a multivariable proportional subdistribution hazards model to estimate adjusted subdistribution hazard ratios (asHRs) and associated 95% CIs. Overall survival was estimated using the Kaplan-Meier method, and the log-rank test was applied to compare survival differences by sex, region, WHO COVID-19 severity stage, and number of comorbidities.

Two-sided *P* < .05 was considered statistically significant. All regression models were applied to the 20 imputed data sets, and estimates were combined according to Rubin rules.^34,35^ All analyses were performed using Stata software, version 16.1 (StataCorp LLC).

## Results

### Demographic Characteristics and Clinical Manifestations at Admission

Data from 469 children and adolescents from central Africa (39 patients [8.3%]), eastern Africa (172 patients [36.7%]), southern Africa (208 patients [44.3%]), and western Africa (50 patients [10.7%]) were analyzed. The age range for the cohort was 3 months to 19 years, with a median age of 5.9 years (IQR, 1.6-11.1 years). Among 468 patients, 223 (47.6%) were female, and 245 (52.4%) were male (Table 1; eTable 3, eFigure 1, and eFigure 2 in Supplement 1). Data on race and ethnicity were not collected. At hospital admission, 246 of 469 patients (52.5%) presented with mild or moderate disease, and 223 of 469 patients (47.5%) presented with severe or critical disease based on WHO severity staging. Most study sites (17 of 25 hospitals [68.0%]) were in urban areas, and almost all study sites (23 of 25 hospitals [92.0%]) had supplemental oxygen available on site (eTable 1 and eFigure 3 in Supplement 1). Of 372 children and adolescents with documented oxygen saturation levels, 78 (21.0%) had levels lower than 95%.

**Table 1.** Demographic Characteristics, Comorbidities, and Outcomes Among Children and Adolescents Hospitalized With COVID-19 by Region of Residence in Africa

Characteristic	No./total No. (%)				
	Total (N = 469)	Eastern Africa (n = 172)	Western Africa (n = 50)	Central Africa (n = 39)^a^	Southern Africa (n = 208)^b^
Age, median (IQR), y	5.9 (1.7–11.1)	9.0 (2.1-14.0)	6.0 (2.5-13.0)	14.0 (9.0-16.0)	2.7 (0.8-8.8)
Sex					
Female	223/468 (47.6)	92/171 (53.8)	23/50 (46.0)	23/39 (59.0)	85/208 (40.9)
Male	245/468 (52.4)	79/171 (46.2)	27/50 (54.0)	16/39 (41.0)	123/208 (59.1)
Outcomes					
No oxygen supplementation	309/452 (68.4)	138/164 (84.1)	32/50 (64.0)	28/33 (84.8)	111/205 (54.1)
Oxygen supplementation	160/463 (34.6)	28/166 (16.9)	18/50 (36.0)	5/39 (12.8)	109/208 (52.4)
ICU admission	69/461 (15.0)	15/164 (9.1)	3/50 (6.0)	5/39 (12.8)	46/208 (22.1)
Any invasive ventilation	34/436 (7.8)	7/148 (4.7)	0	1/33 (3.0)	26/207 (12.6)
Death	39/468 (8.3)	12/172 (7.0)	7/50 (14.0)	2/38 (5.3)	18/208 (8.7)
Hospital discharge	418/468 (89.3)	158/172 (91.9)	42/50 (84.0)	35/38 (92.1)	183/208 (88.0)
Comorbidities					
Asthma	5/455 (1.1)	1/162 (0.6)	0	1/36 (2.8)	3/208 (1.4)
Hypertension (age appropriate)	21/454 (4.6)	0	2/48 (4.2)	0	19/207 (9.2)
Type 1 diabetes	1/360 (0.3)	0	0	0	1/208 (0.5)
Cancer	27/459 (5.9)	2/164 (1.2)	2/49 (4.1)	0	23/208 (11.1)
Chronic kidney disease	10/457 (2.2)	1/165 (0.6)	2/49 (4.1)	0	7/206 (3.4)
Chronic liver disease	3/458 (0.7)	0	1/49 (2.0)	0	2/207 (1.0)
Cardiac disease	25/302 (8.3)	0	2/50 (4.0)	0	22/202 (10.9)
Chronic lung disease	8/343 (2.3)	2/64 (3.1)	1/48 (2.1)	0	5/208 (2.4)
Chronic neurological disorders^c^	22/458 (4.8)	6/164 (3.7)	1/49 (2.0)	0	15/208 (7.2)
Hematological disorders^d^	16/459 (3.5)	6/164 (3.7)	3/49 (6.1)	1/38 (2.6)	6/208 (2.9)
Active tuberculosis	12/444 (2.7)	1/163 (0.6)	0	1/31 (3.2)	10/201 (5.0)
Past tuberculosis	6/434 (1.4)	0	2/49 (4.1)	1/30 (3.3)	3/201 (1.5)
HIV infection	11/342 (3.2)	2/92 (2.2)	2/26 (7.7)	0	7/187 (3.7)
Acute malaria^e^	8/437 (1.8)	4/172 (2.3)	3/18 (16.7)	1/39 (2.6)	0

Abbreviation: ICU, intensive care unit.

^a^ The Central Africa cohort includes 34 children from the Nachega et al^39^ DR Congo study.

^b^ Includes 62 children from the Van der Zalm et al^17^ South African cohort.

^c^ Epilepsy, cerebral palsy, and other.

^d^ Sickle cell anemia, thalassemia, and glucose-6-phosphate dehydrogenase deficiency.

^e^ No data reported for Nigeria; western Africa data were analyzed for Ghana only.

The most frequently documented symptoms of SARS-CoV-2 infection were cough (170 of 460 patients [37.0%]), fever (143 of 461 patients [31.0%]), rhinorrhea (116 of 463 patients [25.1%]), and respiratory distress (76 of 328 patients [23.2%]). Eighteen of 297 cases (6.1%) were clinically suspected (6 patients) or confirmed (12 patients) as MIS-C (eTable 3 in Supplement 1). A total of 115 of 469 patients (24.5%) had at least 1 preexisting medical condition at admission, including cancer (27 of 459 patients [5.9%]), hypertension (21 of 454 patients [4.6%]), chronic kidney disease (10 of 457 patients [2.2%]), chronic neurological disorders (22 of 458 patients [4.8%]), cardiac disease (25 of 302 patients [8.3%]), chronic lung disease (8 of 343 patients [2.3%]), hematological disorders (16 of 459 patients [3.5%]), HIV infection (11 of 342 patients [3.2%]), and active tuberculosis (12 of 444 patients [2.7%]) (Table 1 and eTable 4 in Supplement 1). All patients received supportive treatment per WHO recommendations,^31^ but no experimental therapeutic medications (eg, remdesivir or interleukin 6 receptor blockade with tocilizumab) for the treatment of COVID-19 were locally available.

At the time of data extraction, among 468 children and adolescents with complete data on outcomes, 418 patients (89.3%; 95% CI, 86.2%-92.0%) were discharged from the hospital, 39 patients (8.3%; 95% CI, 6.0%-11.2%) died, 16 patients (3.4%; 95% CI, 2.0%-5.5%) remained hospitalized (with 11 patients [2.4%; 95% CI, 1.2%-4.2%] remaining hospitalized at 2-40 days [median, 18 days; IQR, 7-24 days] after admission), and 1 patient (0.2%; 95% CI, 0%-1.2%) had missing outcome data. Among 69 patients admitted to the ICU, 22 (31.9%) died. Of the 39 total deaths, information on the presence or absence of clinical features of MIS-C was available for 26 patients (66.7%); among those, 4 patients (15.4%; 22.2% of the 18 patients with suspected or confirmed MIS-C) had confirmed or suspected MIS-C. Twelve of the 39 deaths (30.8%) occurred among the 78 children who were younger than 1 year.

### Primary Outcome

**Figure 1.**
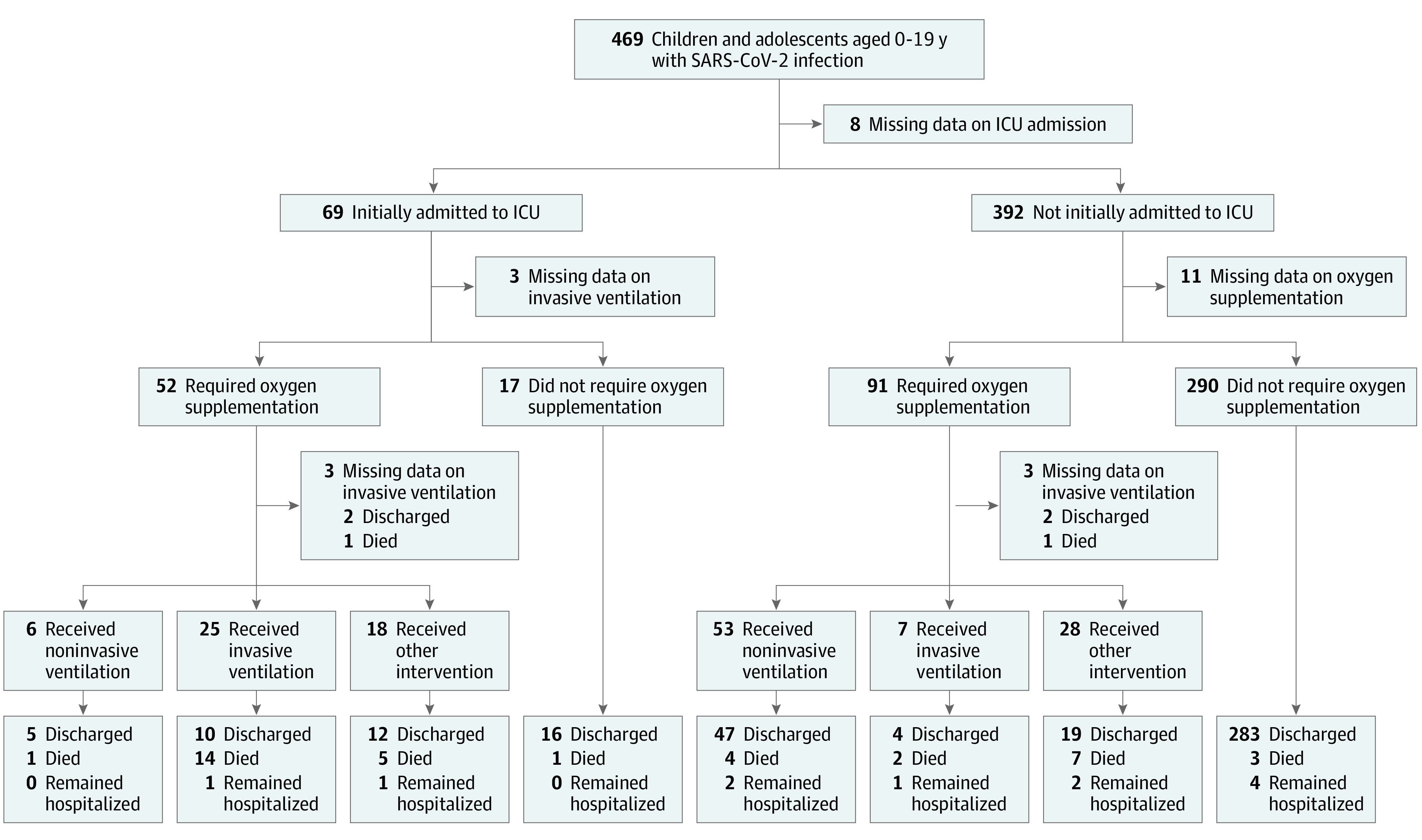
Flow Diagram of Clinical Outcomes Stratified by Initial Intensive Care Unit Admission and Oxygen Supplementation ICU indicates intensive care unit.

**Figure 2.**
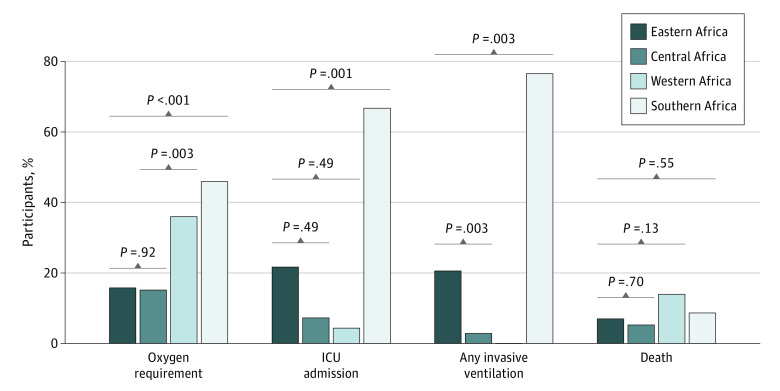
Clinical Outcomes of Children and Adolescents With COVID-19 by Region A total of 26 patients (15.8%) in eastern Africa, 5 patients (15.2%) in central Africa, 18 patients (36.0%) in western Africa, and 94 patients (45.9%) in southern Africa required oxygen supplementation. A total of 15 patients (21.7%) in eastern Africa, 5 patients (7.3%) in central Africa, 3 patients (4.4%) in western Africa, and 46 patients (66.7%) in southern Africa were admitted to the intensive care unit (ICU). A total of 7 patients (20.6%) in eastern Africa, 1 patient (2.9%) in central Africa, 0 patients in western Africa, and 26 patients (76.5%) in southern Africa required invasive mechanical ventilation. A total of 12 patients (7.0%) in eastern Africa, 2 patients (5.3%) in central Africa, 7 patients (14.0%) in western Africa, and 18 patients (8.7%) in southern Africa died.

Among those with complete information, 160 of 463 patients (34.6%) were either admitted to the ICU (69 of 461 patients [15.0%]) or required supplemental oxygen (143 of 452 patients [31.6%]). A total of 76 of 379 patients (20.1%) received noninvasive respiratory support via high-flow nasal cannula, and 34 of 436 patients (7.8%; 34 of 160 patients [21.2%] admitted to the ICU) required invasive mechanical ventilation. Overall, the clinical disease severity outcome among 469 patients was distributed as follows: 305 patients (65.0%) had mild severity, 72 (15.4%) received supplemental oxygen, 18 (3.8%) received mechanical ventilation, and 39 (8.3%) died. The southern region of Africa had the highest proportion of patients requiring ICU admission and/or oxygen supplementation (109 of 208 patients [52.4%]). The sequence of events from hospital admission to clinical outcomes is shown in Figure 1, and overall clinical outcomes by region are shown in Table 1, Figure 2, and eTable 3 in Supplement 1.

**Figure 3.**
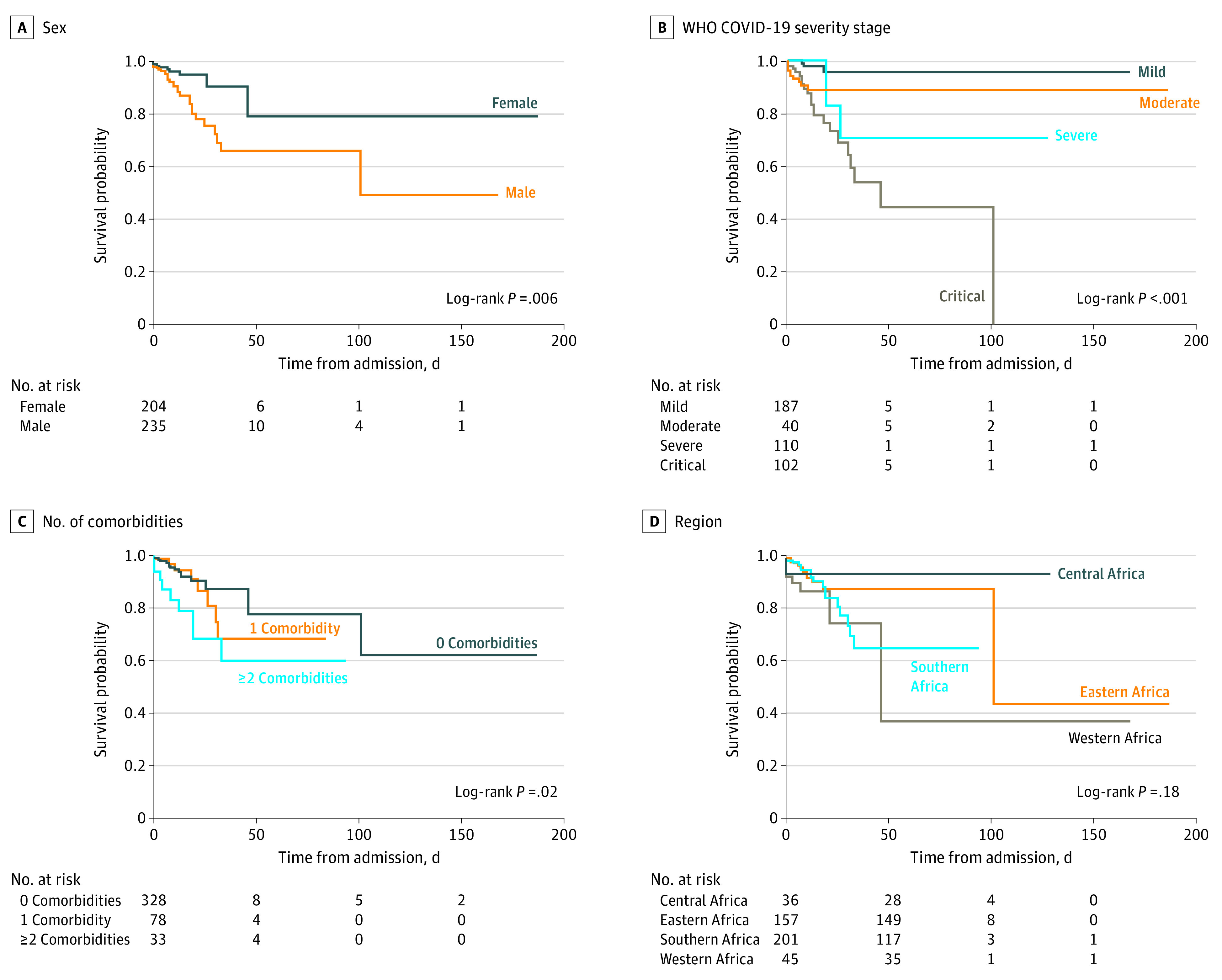
Kaplan-Meier Curves Comparing Survival Differences by Sex, World Health Organization COVID-19 Severity Stage, Number of Comorbidities, and Region A, The hazard ratio (HR) was 2.77 (95% CI, 1.30-5.88; *P* = .008) for male patients compared with female patients (reference group). B, The HRs were 3.27 (95% CI, 0.65-16.38; *P* = .15) for moderate stage disease, 5.90 (95% CI, 1.62-21.49; *P* = .007) for severe stage disease, and 10.68 (95% CI, 3.18-35.89; *P* = .001) for critical stage disease compared with mild stage disease (reference group). C, The HRs were 1.37 (95% CI, 0.59-3.15; *P* = .46) for 1 comorbidity and 2.89 (95% CI, 1.28-6.52; *P* = .01) for 2 or more comorbidities compared with 0 comorbidities (reference group). D, The HRs were 0.60 (95% CI, 0.13-2.70; *P* = .50) for central Africa, 1.29 (95% CI, 0.61-2.74; *P* = .50) for southern Africa, and 2.35 (95% CI, 0.92-6.00; *P* = .07) for western Africa compared with eastern Africa (reference group). WHO indicates World Health Organization.

In the unadjusted analyses, male sex (hazard ratio [HR], 2.77; 95% CI, 1.31-5.89; *P* = .008), the presence of 2 or more comorbidities (HR, 2.89; 95% CI, 1.28-6.52; *P* = .01), WHO COVID-19 severe disease stage (HR, 5.91; 95% CI, 1.62-21.49; *P* = .007), and WHO COVID-19 critical disease stage (HR, 10.68; 95% CI, 3.18-35.89; *P* < .001) were associated with higher risk of death. There were no significant regional differences in the risk of death (Figure 3). In multivariable ordinal logistic regression analyses, the likelihood of more severe vs less severe outcomes among children younger than 1 year was 4.89 (95% CI, 1.44-16.61; *P* = .01) times higher than that among adolescents aged 15 to 19 years. The presence of hypertension (aOR, 5.91; 95% CI, 1.89-18.50; *P* = .002), chronic lung disease (aOR, 2.97; 95% CI, 1.65-5.37; *P* < .001), or a hematological disorder (aOR, 3.10; 95% CI, 1.04-9.24; *P* = .04) were also independently associated with more severe outcomes. The presence of HIV infection (aOR, 2.02; 95% CI, 0.97-4.20; *P* = .06) was not associated with more severe outcomes (Table 2).

**Table 2.** Univariable and Multivariable Ordinal Logistic Regression Model Using 5 Levels of COVID-19 Disease Severity as Primary Outcome^a^

Characteristic	Unadjusted OR (95% CI)	*P* value	Adjusted OR (95% CI)	*P* value
Age group, y				
<1	5.23 (2.07-13.19)	<.001	4.89 (1.44-16.61)	.01
1-4	1.46 (0.62-4.16)	.01	1.46 (0.61-3.47)	.39
5-9	1.42 (0.62-3.22)	.40	1.04 (0.35-3.14)	.94
10-14	1.07 (0.44-2.63)	.88	0.90 (0.27-2.99)	.86
15-19	1 [Reference]	NA	1 [Reference]	NA
Sex				
Male	1.54 (0.93-2.54)	.09	1.14 (0.81-1.61)	.44
Female	1 [Reference]	NA	1 [Reference]	NA
Region				
Eastern Africa	1 [Reference]	NA	1 [Reference]	NA
Western Africa	2.43 (0.33-17.69)	.38	0.59 (0.22-1.60)	.31
Central Africa	0.68 (0.07-6.84)	.74	0.48 (0.13-1.80)	.28
Southern Africa	4.31 (0.78-23.71)	.09	0.83 (0.59-1.16)	.28
WHO COVID-19 stage^b^				
Mild	1 [Reference]	NA	NA	NA
Moderate	2.91 (0.83-10.20)	.10	NA	NA
Severe	5.76 (2.12-15.65)	.001	NA	NA
Critical	49.20 (21.38-113.23)	<.001	NA	NA
Type of comorbidity				
No hematologic disorders	1 [Reference]	NA	1 [Reference]	NA
Asthma	5.39 (1.36-21.32)	.02	3.84 (0.81-18.06)	.09
Hypertension	5.86 (2.20-15.66)	<.001	5.91 (1.89-18.50)	.002
Cancer	1.70 (0.75-3.86)	.21	NA	NA
Chronic kidney disease	3.31 (0.21-53.40)	.40	NA	NA
Heart disease	2.26 (0.87-5.87)	.09	1.73 (0.73-4.08)	.21
Chronic lung disease	2.76 (1.40-5.42)	.003	2.97 (1.65-5.37)	<.001
Chronic neurological disorders	1.93 (1.00-3.72)	.05	1.08 (0.63-1.84)	.79
Hematologic disorders	3.03 (1.02-9.01)	.047	3.10 (1.04-9.24)	.04
Epilepsy	1.24 (0.56-2.76)	.59	NA	NA
Current tuberculosis	1.41 (0.50-3.95)	.52	NA	NA
Past tuberculosis	2.45 (0.63-9.55)	.20	NA	NA
HIV-positive status	2.18 (0.79-3.51)	.13	2.02 (0.97-4.20)	.06
No. of comorbidities				
0	1 [Reference]	NA	1 [Reference]	NA
1	2.22 (1.19-4.13)	.01	1.95 (1.08-3.50)	.03
≥2	3.49 (1.64-7.45)	.001	3.75 (1.71-8.22)	.001

Abbreviations: ICU, intensive care unit; NA, not applicable; OR, odds ratio; WHO, World Health Organization.

^a^ The 5 levels of COVID-19 disease severity were no oxygen supplementation, oxygen supplementation, ICU admission, mechanical ventilation, and death.

^b^ The analysis did not adjust for WHO COVID-19 stage because its components were associated with the primary ordinal outcome.

### Secondary Outcome: Length of Hospital Stay

The median length of hospital stay was 9 days (IQR, 5-16 days) among patients who recovered and 8 days (IQR, 3-19 days) among those who died. Among 10 patients (1 with missing data) who remained hospitalized at the time of data collection, the median length of hospital stay was 18 days (IQR, 7-24 days). In the adjusted competing-risk analysis of time to discharge, age younger than 1 year (asHR, 0.48; 95% CI, 0.27-0.87; *P* = .02), the presence of 1 comorbidity (asHR, 0.54; 95% CI, 0.40-0.72; *P* < .001), and the presence of 2 or more comorbidities (asHR, 0.26; 95% CI, 0.18-0.38; *P* < .001) were associated with reduced rates of hospital discharge. The rate of discharge among children and adolescents living in southern Africa was significantly higher compared with those living in eastern Africa (asHR, 2.04; 95% CI, 1.27-3.26; *P* = .003). Discharge rates in western Africa (asHR, 1.05; 95% CI, 0.58-1.90; *P* = .87) and central Africa (asHR, 0.82; 95% CI, 0.45-1.49; *P* = .52) were comparable with those in eastern Africa.

## Discussion

This multicountry cohort study of pediatric COVID-19 in sub-Saharan Africa revealed relatively high morbidity and mortality, with greater likelihood of more severe outcomes among children younger than 1 year and those with hypertension, chronic lung disease, or a hematologic disorder. Furthermore, in a competing-risk analysis of time to discharge, age younger than 1 year, the presence of 1 comorbidity, and the presence of 2 or more comorbidities were independently associated with reduced rates of hospital discharge. Overall, 34.6% of hospitalized children and adolescents were admitted to the ICU or required oxygen supplementation, and 21.2% of those admitted to the ICU required invasive ventilation. The region with the highest proportion of children and adolescents requiring ICU admission and/or oxygen supplementation (52.4%) was southern Africa, where there was better availability of high-quality critical care than in other sub-Saharan African countries.^25^ The proportion of children and adolescents requiring ICU admission or oxygen supplementation in this study was similar to or higher than the proportions reported in studies of non-African countries but was likely underestimated because of the limited availability of pediatric ICUs in much of sub-Saharan Africa.^36,37,38^ Of note, our study included 62 hospitalized patients from what was previously the largest (N = 159) cohort of African children with COVID-19.^17^ In that study, 11 of 51 hospitalized children (21.6%) required ICU admission; of those, 4 children required mechanical ventilation, but none died.

Overall, 8.3% of inpatients in the present study died. In comparison, among 766 patients with COVID-19 from a previous Nachega et al^39^ study of the Democratic Republic of the Congo, in-hospital mortality among those younger than 20 years was 11.8% (4 of 34 patients), all of which occurred among adolescents; this hazard of death was almost 7 times that of adults aged 20 to 39 years (mortality rate, 2.4% [6 of 248 adults]; aHR, 6.62 [95% CI, 1.85-23.65; *P* = .004]). A South African surveillance study of childhood deaths identified SARS-CoV-2 infection in antemortem and/or postmortem sampling among 11.7% of 171 children who died, and 90% of SARS-CoV-2–associated deaths were among infants.^40^ This finding was consistent with our finding of higher frequency of severe outcomes, including death, among infants and with the results of a study conducted by Oliveira et al^41^ in Brazil. In a global systematic review of severe pediatric COVID-19 illness, Kitano et al^15^ also reported that infants had the highest mortality, and the overall case fatality rate was significantly higher in low- and middle-income countries (0.24%) than in high-income countries (0.01%). In our study, mortality was high after ICU admission (31.9%) and substantially greater than the 0% to 0.5% mortality observed in pediatric studies conducted in high-resource settings^1,4,5,6,15^ but closer to the mortality (approximately 50%) reported in the African COVID-19 Critical Care Outcomes Study involving adults.^22^

We also found that hypertension, chronic lung diseases, and hematologic disorders were independently associated with severe clinical outcomes, including death. Preexisting comorbidities have been associated with worse COVID-19 prognosis in children and adults in other studies.^41,42,43,44,45^ In 1 study,^45^ among 43 465 US children diagnosed with COVID-19 from March 2020 to January 2021, 28.7% had underlying medical conditions; the most important risk factors associated with hospitalization or severe COVID-19 were type 1 diabetes, obesity, cardiac or circulatory congenital anomalies, hypertension, neuropsychiatric disorders, and complex chronic disease. In our study as well as the Oliveira et al^41^ study from Brazil, an increase in COVID-19–associated mortality occurred as the number of preexisting comorbidities increased.

In this study, HIV infection was not associated with worse outcome severity, possibly because of low numbers of children living with HIV. This finding necessitates further research. Of note, published data on the association of HIV infection with COVID-19 outcomes among adults has been inconsistent.^46,47,48^ However, a recent data review by the WHO found that HIV infection in adults was a risk factor associated with severe and critical illness at hospital admission and in-hospital mortality after adjusting for age, sex, and underlying conditions.^49^

Our findings have several implications for clinical practice or health policy. The high morbidity and mortality among hospitalized African children and adolescents with comorbidities suggest that targeting these populations for prompt COVID-19 vaccination may be warranted when vaccines become available. Therapeutic interventions should be specifically evaluated among children and adolescents with severe COVID-19 illness and made available as appropriate. In addition, limitations in the quality and scope of pediatric general and critical care services in Africa need to be addressed to improve outcomes among children and adolescents with severe COVID-19 illness and other serious health conditions.

### Limitations

This study has several limitations. Our findings of higher COVID-19–associated in-hospital mortality among children and adolescents in sub-Saharan Africa compared with those in non-African settings needs to be interpreted with consideration of important factors. First, we studied only hospitalized children and adolescents, whereas most published studies from China, Europe, and the US^1,2,3,4,5,6,7,8,9,15^ included hospitalized, nonhospitalized, and asymptomatic patients. Our data are not generalizable to outpatient populations. In addition, because of limited hospital resources in sub-Saharan Africa, there may be higher thresholds for hospitalization compared with those in more resource-rich settings, potentially producing a cohort of inpatients who had more severe illness.

Second, a high prevalence of concurrent endemic infections and noncommunicable diseases, malnutrition, and associated dietary deficiencies may have had implications for COVID-19 outcomes in sub-Saharan Africa. Third, MIS-C did not appear to be a major factor associated with mortality in our study; of 26 evaluable deaths, only 4 (15.4%) had confirmed or suspected MIS-C. However, MIS-C cases were likely underestimated owing to the limited availability of tests (eg, tests for inflammatory biomarkers).

Fourth, the limited availability of essential equipment and the narrower scope of pediatric intensive care (compared with adult care) in sub-Saharan Africa likely had implications for the high mortality observed in the present study cohort.^21,25^ The finding of greater use of intensive care, oxygen supplementation, and mechanical ventilation in the southern African region vs other regions as well as the lower risk of in-hospital death in this region likely reflected varied availability of resources across African regions. Furthermore, these results highlight the opportunity for improved outcomes afforded by greater availability of high-quality pediatric intensive care.

Fifth, the retrospective study design relied on record extraction of routinely collected and available data; however, fewer than 6% of extracted outcome data were missing, and we used multiple imputation techniques for missing data on comorbidities to minimize biased OR estimates. Sixth, limited availability of laboratory tests and diagnostic procedures may have produced underdiagnosis of COVID-19 and some associated features (eg, MIS-C) and precluded reporting and further analysis of immunological status among children living with HIV infection.

Seventh, our lack of a SARS-CoV-2–negative comparator group and the general limited access to diagnostic testing prevents us from drawing conclusions about the relative prevalence and severity of COVID-19 vs other pediatric diseases in sub-Saharan Africa. In addition, statistical modeling of outcomes for each region was limited by small numbers within regions. However, the power of our study comes from pooling data across health facilities and regions as well as adjustment for any potential facility-level differences that may have had consequences for outcomes.

## Conclusions

In this cohort study of 6 countries in sub-Saharan Africa, morbidity and mortality rates among hospitalized children and adolescents with COVID-19 were substantially higher than those reported in non-African settings and were associated with age younger than 1 year and select noncommunicable disease comorbidities. These findings provide new data that may be used to inform pediatric COVID-19 health policy in Africa. With hundreds of millions of African children and adolescents at risk of adverse outcomes, COVID-19 vaccination and therapeutic interventions are much needed for this population.

## Notes

Supplement 1. **eTable 1.** Participating Health Facilities **eTable 2.** Research Ethics Committee Approvals **eTable 3.** Demographic Characteristics and Comorbidities at Admission **e-Table 4.** Demographic Characteristics, Comorbidities, and Outcomes Among Hospitalized Children and Adolescents With COVID-19 by Region of Residence in Africa **eFigure 1.** Map of Africa Displaying Participating Countries and Number of Study Sites **eFigure 2.** Bar Graph of Number of Children and Adolescents With COVID-19 by Country **eFigure 3.** Distribution of Pediatric Services by Region

Supplement 2. Nonauthor Collaborators. **Members of the African Forum for Research and Education in Health (AFREhealth) COVID-19 Research Collaboration on Children and Adolescents**
