# Supplementary material for: Assessment of Clinical Outcomes Among Children and Adolescents Hospitalized With COVID-19 in 6 Sub-Saharan African Countries
Source: JAMA Pediatr. 2022 Jan 19;176(3):e216436. doi: 10.1001/jamapediatrics.2021.6436 (PMC8771438; doi:10.1001/jamapediatrics.2021.6436)
Supplement: Supplement 1. — eTable 1. Participating Health Facilities eTable 2. Research Ethics Committee Approvals eTable 3. Demographic Characteristics and Comorbidities at Admission e-Table 4. Demographic Characteristics, Comorbidities, and Outcomes Among Hospitalized Children and Adolescents With COVID-19 by Region of Residence in Africa eFigure 1. Map of Africa Displaying Participating Countries and Number of Study Sites eFigure 2. Bar Graph of Number of Children and Adolescents With COVID-19 by Country eFigure 3. Distribution of Pediatric Services by Region [file jamapediatr-e216436-s001.pdf]

Supplemental Online Content

Nachega JB, Sam-Agudu NA, Machekano RN, et al; African Forum for Research and Education in Health (AFREhealth) COVID-19 Research Collaboration on Children and Adolescents. Assessment of clinical outcomes among children and adolescents hospitalized with COVID-19 in 6 sub-Saharan African countries. *JAMA Pediatr*. Published online January 19, 2022. doi:10.1001/jamapediatrics.2021.6436

eTable 1. Participating Health Facilities

eTable 2. Research Ethics Committee Approvals

eTable 3. Demographic Characteristics and Comorbidities at Admission

eTable 4. Demographic Characteristics, Comorbidities, and Outcomes Among Hospitalized Children and Adolescents With COVID-19 by Region of Residence in Africa

eFigure 1. Map of Africa Displaying Participating Countries and Number of Study Sites

eFigure 2. Bar Graph of Number of Children and Adolescents With COVID-19 by Country

eFigure 3. Distribution of Pediatric Services by Region

This supplementary material has been provided by the authors to give readers additional information about their work.

© 2022 Nachega JB et al. *JAMA Pediatrics*.

eTable 1. Participating Health Facilities (N = 25)

Characteristic	Total 6 countries N= 25 facilities		West Africa (Ghana, Nigeria) N= 4 facilities		Central Africa (DR Congo) N=7 facilities		East Africa (Uganda, Kenya) N= 4 facilities		Southern Africa (South Africa) N= 10 facilities	
	n or average	%	n or average	%	n or average	%	n or average	%	n or average	%
**Level of Care**										
Primary	4	16.0	-	-	1	14.3	-	-	3	30.0
Secondary	12	48.0	1	25.0	5	71.4	2	50.0	4	40.0
Tertiary	8	32.0	3	75.0	1	14.3	2	50.0	2	20.0
Other (Quaternary)	1	4.0	-	-	-		-	-	1	10.0
**Funding**										
Public (Government)	21	84.0	4	100.0	3	42.9	4	100.0	10	100.0
Private	3	12.0	-	-	3	42.9	-	-	-	-
Public-Private	1	4.0	-	-	1	14.3	-	-	-	-
**Location Type**										
Urban	17	68.0	3	75.0	7	100.0	4	100.0	3	30.0
Peri-Urban	6	24.0	1	25.0	-	-	-	-	5	50.0
Rural	2	8.0	-	-	-	-	-	-	2	20.0
**General pediatric services**										
Average no. of general pediatric ward beds	37 (N*= 24)	N/A	30	N/A	31	N/A	101 (N*= 3)	N/A	31	N/A
Average no. of doctors per 8 hr shift	3 (N*= 17)	N/A	4	N/A	2	N/A	4 (N*= 3)	N/A	2	N/A
Average no. of nurses per 8 hr shift	4 (N*= 17)	N/A	5	N/A	3	N/A	5 (N*= 3)	N/A	3	N/A
**PICU services**										
Number of facilities with on-site PICU	11	44.0	2	50.0	5	71.4	1	25.0	3	30.0
Average no. of PICU beds (among facilities with on-site PICU)	7	N/A	4	N/A	8	N/A	6	N/A	9	N/A
Average no. of doctors per 8 hr shift	3 (N*= 6)	N/A	3	N/A	2	N/A	3	N/A	3	N/A
Average no. of nurses per 8 hr shift	5 (N*= 6)	N/A	3	N/A	2	N/A	2	N/A	7	N/A
**On-site oxygen supply**										
**On-site oxygen supply available?**	23	92.0	4	100.0	5	71.4	4	100.0	10	100.0
**Consistency of oxygen supply**	(N*=23)									
Highly consistent (100%)	18	78.3	3	75.0	1	20.0	4	100.0	10	100.0
>75% of the time	2	8.7	1	25.0	1	20.0	-	-	-	-
50-75% of the time	2	8.7		-	2	40.0	-	-	-	-
<50% of the time	1	4.3		-	1	20.0	-	-	-	-
**No. of facilities with on-site mechanical ventilators**	14	56.0	2	50.0	2	28.6	2	50.0	8	80.0
**Average number of functional mechanical ventilators** (among facilities with on-site ventilators)	4	N/A	3	N/A	3	N/A	7	N/A	4	N/A

N/A: not applicable; PICU: pediatric intensive care unit; N: applicable facility denominator for this question; N/A: not applicable*

© 2022 Nachega JB et al. *JAMA Pediatrics*.

eTable 2. Research Ethics Committee Approvals

Country	Facility Name	Approval Number	Date Approved
	**Region 1: West Africa**		
**Ghana**	Cape Coast Teaching Hospital	CCTHERC/EC/2020/104	19 November 2020
	Komfo Anokye Teaching Hospital	KATH IRB/AP/020/21	12 March 2021
**Nigeria**	Ahmadu Bello University Teaching Hospital	ABUTH/HREC/CL/05	30 October 2020
	Asokoro District Hospital	FCTA/HHSS/HMB/ADH/057/20	19 November 2020
	**Region 2: Central Africa**		
**Democratic Republic of the Congo**	Cliniques Universitaires de Kinshasa	UNIKIN/ESP/CE/64/2021	17 July 2020
	Clinique Ngaliema	UNIKIN/ESP/CE/64/2021	17 July 2020
	Hôpital de l’Amitié Sino-Congolaise	UNIKIN/ESP/CE/64/2021	17 July 2020
	Centre Hospitalier Vijana	UNIKIN/ESP/CE/64/2021	17 July 2020
	Centre Médical de Kinshasa	UNIKIN/ESP/CE/64/2021	17 July 2020
	Hôpital Saint Joseph	UNIKIN/ESP/CE/64/2021	17 July 2020
	Hôpital Monkolé	UNIKIN/ESP/CE/64/2021	17 July 2020
	**Region 3: East Africa**		
**Kenya**	Kenyatta National Hospital	KNH-ERC/A/386	2 November 2020
**Uganda**	Mulago National Referral Hospital	MNRH/REC/16/12/2020	16 December 2020
	Entebbe Regional Referral Hospital	ERRH/19/11/2020	19 November 2020
	Kawempe National Regional Referral Hospital	KNRH1/11/20	01 November 2020
	**Region 4: Southern Africa**		
**South Africa**	Tygerberg Teaching Hospital	N20/04/013_COVID-019	04 February 2021
	Khayelitsha District Hospital	N20/04/013_COVID-019	04 February 2021
	King Edward VIII Hospital	BREC/00002196/2020	03 March 2021
	Prince Mshiyeni Memorial Hospital	BREC/00002196/2020	03 March 2021
	Port Shepstone Hospital	BREC/00002196/2020	03 March 2021
	St. Mary’s Hospital	BREC/00002196/2020	03 March 2021
	Inkosi Albert Luthuli Central Hospital	BREC/00002196/2020	03 March 2021
	Mahatma Gandhi Memorial Hospital	BREC/00002196/2020	03 March 2021
	Murchinson Hospital	BREC/00002196/2020	03 March 2021
	RK Khan Hospital	BREC/00002196/2020	03 March 2021

© 2022 Nachega JB et al. *JAMA Pediatrics*.

eTable 3. Demographic Characteristics and Comorbidities at Admission (N = 469)

Characteristic	No.	%
**Median age, years [IQR]**	5.9 [1.6 – 11.1]	
**Age group**		
<1 year	79	16.8
1 - 4 years	134	28.6
5 - 9 years	83	17.7
10 - 14 years	103	22.0
15 - 19 years	70	14.9
**Sex**		
Male	245	52.4
Female	223	47.6
Missing	1	
**Country**		
DRC*	39	8.3
Ghana	18	3.8
Kenya	70	14.9
Nigeria	32	6.8
South Africa**	208	44.3
Uganda	102	21.7
**WHO COVID-19 staging**		
Mild	203	43.3
Moderate	43	9.2
Severe	116	24.7
Critical	107	22.8
**Signs and symptoms at admission**		
Cough	170/460	37
Fever	143/461	31.0
Rhinorrhea	116/463	25.1
Respiratory distress	76/328	23.2
Oxygen saturation <95%	78/372	21.0
Vomiting	88/456	19.3
Fatigue or malaise	80/455	17.6
Chest pain	60/428	14.0
Abdominal pain	61/443	13.8
Irritability	42/461	9.1
Cold hands or feet	30/317	9.5
Headache	40/435	9.2
Rash	31/453	6.8
Sore throat	28/445	6.3
MIS-C	18/297	6.1
Toxic shock syndrome	13/428	3.0
Loss of smell or taste	9/429	2.1
Swollen joints	6/332	1.8

**This includes 34 children from the DR Congo cohort in Nachega et al [39]*
***This includes 62 children from the South African cohort in Van der Zalm et al [17].*
IQR: interquartile range; DRC: the Democratic Republic of the Congo. WHO: World Health Organization; MIS-C: multisystem inflammatory syndrome in children.

© 2022 Nachega JB et al. *JAMA Pediatrics*.

eTable 4. Demographic Characteristics, Comorbidities, and Outcomes among Hospitalized Children and Adolescents with COVID-19 by Region of Residence in Africa

Characteristic	East Africa (N=172)		West Africa (N=50)		Central Africa (N=39)		Southern Africa (N=208)		Total (N=469)	
	n/N	%	n/N	%	n/N	%	n/N	%	n/N	%
Age, median yrs. (IQR)	9.0 (2.1-14)		6.0 (2.5-13)		14.0 (9.0-16.0)		2.7 (0.8-8.8)		5.9 (1.7–11.1)	
**Outcomes**										
ICU admission	15/164	9.1	3/50	6.0	5/39	12.8	46/208	22.1	69/461	15.0
ICU or oxygen supplement	28/166	16.9	18/50	36.0	5/39	12.8	109/208	52.4	160/463	34.6
Any invasive ventilation	7/148	4.7	0/48	0.0	1/33	3.0	26/207	12.6	34/436	7.8
Died	12/172	7.0	7/50	14.0	2/38	5.3	18/208	8.7	39/468	8.3
Discharged	158/172	91.9	42/50	84.0	35/38	92.1	183/208	88.0	418/468	89.3
**Comorbidities**										
Asthma	1/162	0.6	0/49	0.0	1/36	2.8	3/208	1.4	5/455	1.1
Hypertension (age appropriate)	0/164	0.0	2/48	4.2	0/35	0.0	19/207	9.2	21/454	4.6
Current tuberculosis	1/163	0.6	0/49	0.0	1/31	3.2	10/201	5.0	12/444	2.7
Past tuberculosis	0/154	0.0	2/49	4.1	1/30	3.3	3/201	1.5	6/434	1.4
Type 1 diabetes mellitus	0/66	0.0	0/48	0.0	0/38	0.0	1/208	0.5	1/360	0.3
Malignancy	2/164	1.2	2/49	4.1	0/38	0.0	23/208	11.1	27/459	5.9
Chronic kidney disease	1/165	0.6	2/49	4.1	0/37	0.0	7/206	3.4	10/457	2.2
Chronic liver disease	0/164	0.0	1/49	2.0	0/38	0.0	2/207	1.0	3/458	0.7
Cardiac disease	0/100	0.0	2/50	4.0	0/39	0.0	22/202	10.9	25/302	8.3
Chronic lung disease	2/64	3.1	1/48	2.1	0/23	0.0	5/208	2.4	8/343	2.3
Chronic neurological disorder *	6/164	3.7	1/49	2.0	0/37	0.0	15/208	7.2	22/458	4.8
Hematological disorders**	6/164	3.7	3/49	6.1	1/38	2.6	6/208	2.9	16/459	3.5
HIV Infection	2/92	2.2	2/26	7.7	0/37	0.0	7/187	3.7	11/342	3.2

**Epilepsy, cerebral palsy, and other*
***Sickle cell anemia, thalassemia, and glucose-6-phosphate dehydrogenase deficiency*

© 2022 Nachega JB et al. *JAMA Pediatrics*.

eFigure 1. Map of Africa Displaying Participating Countries and Number of Study Sites

© 2022 Nachega JB et al. *JAMA Pediatrics*.

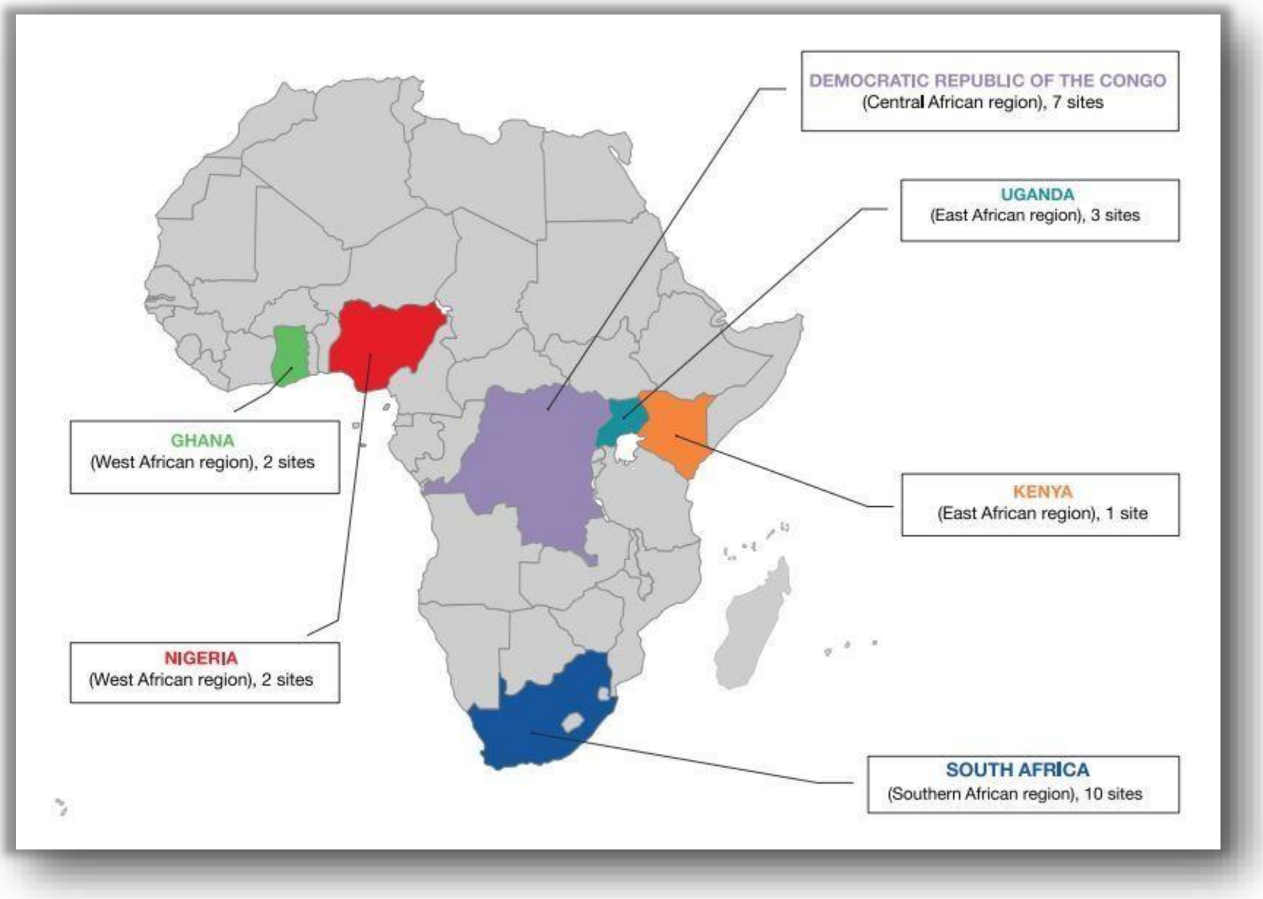

eFigure 2. Bar Graph of Number of Children and Adolescents with COVID-19 by Country (N = 469)

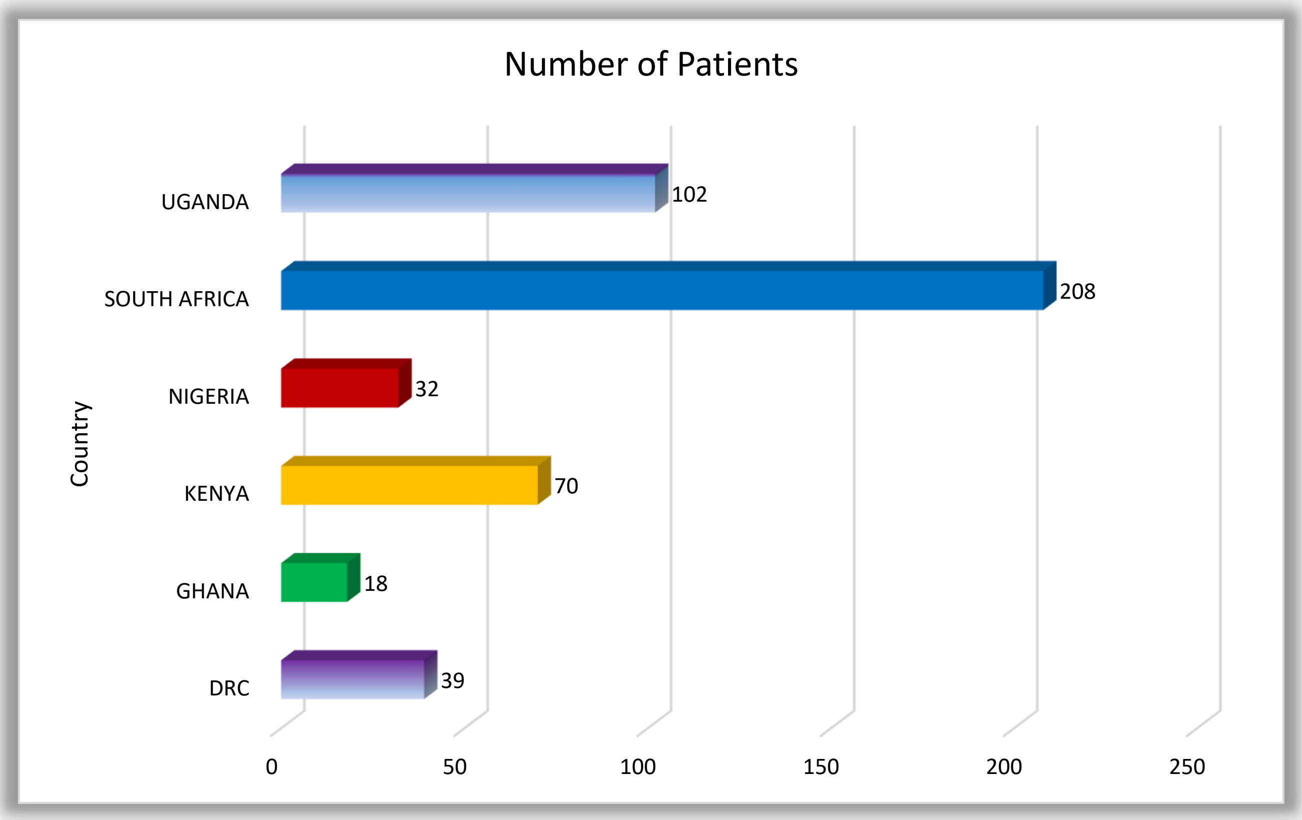

© 2022 Nachega JB et al. *JAMA Pediatrics*.

eFigure 3. Distribution of Pediatric Services by Region

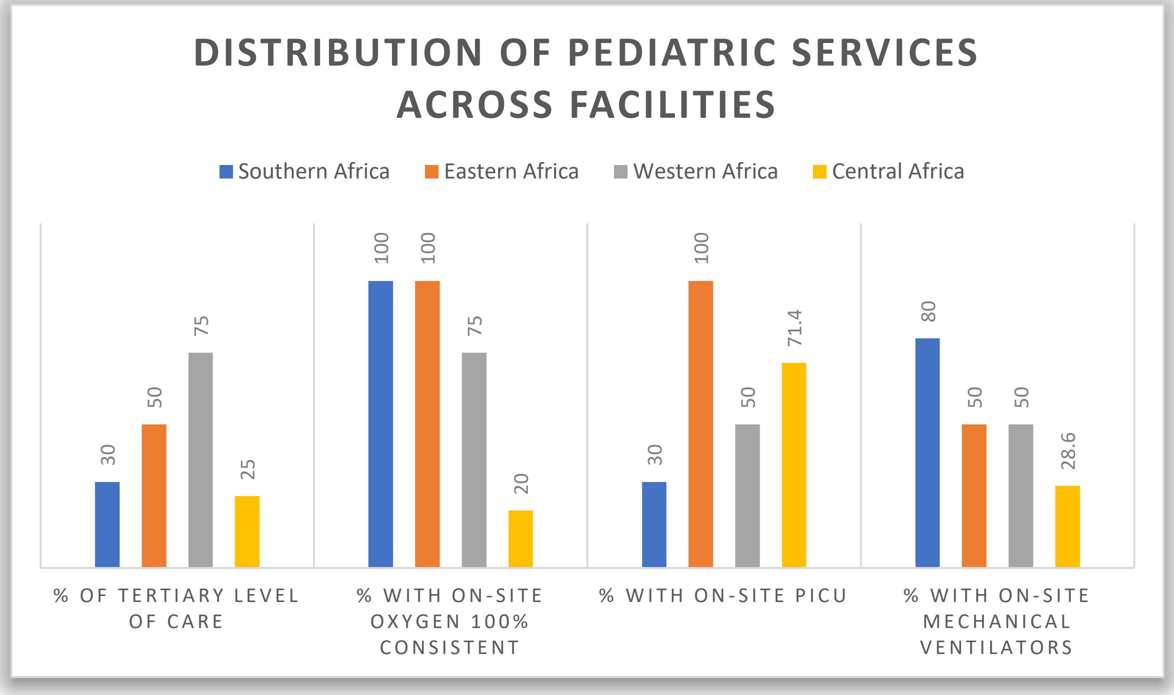

© 2022 Nachega JB et al. *JAMA Pediatrics*.
