# Supplementary material for: Assessment of Clinical Outcomes Among Children and Adolescents Hospitalized With COVID-19 in 6 Sub-Saharan African Countries
Source: JAMA Pediatr. 2022 Jan 19;176(3):e216436. doi: 10.1001/jamapediatrics.2021.6436 (PMC8771438; doi:10.1001/jamapediatrics.2021.6436)
Supplement: Supplement 2. — Nonauthor Collaborators. Members of the African Forum for Research and Education in Health (AFREhealth) COVID-19 Research Collaboration on Children and Adolescents [file jamapediatr-e216436-s002.pdf]

Supplemental Online Content: Nonauthor Collaborators

*Indicates required information. Only first name, last name, and suffix will appear in PubMed.

*Group Name(s): AFREhealth COVID-19 Research Collaboration on Children and Adolescents							
***First Name and Middle Initial(s)**	***Last Name**	***Suffix (eg, Jr, III)**	Academic Degrees	Institution	Location (city, state/province, country)	Role or Contribution, eg, chair, principal investigator	Group (if more than 1 Group listed in the byline) and/or Subgroup (eg, Steering Committee)
Jean B.	Nachega		MD, PhD, MPH	Department of Medicine and Centre for Infectious Diseases, Stellenbosch University Faculty of Medicine and Health Sciences	Cape Town, South Africa	Principal Investigator; Author	
Nadia A.	Sam-Agudu		MD, CTropMed	International Research Center of Excellence, Institute of Human Virology Nigeria	Abuja, Nigeria	Author	
Rhoderick N.	Machekano		PhD, MPH	Division of Epidemiology and Biostatics, Department of Global Health, Stellenbosch University Faculty of Medicine and Health Sciences	Cape Town, South Africa	Author	
Helena	Rabie		MBChB, PhD	Department of Pediatrics and Child Health, Stellenbosch University	Cape Town, South Africa	Author	
Marieke M.	Van der Zalm		MBChB, PhD	Desmond Tutu TB Centre, Department of Pediatrics and Child Health, Stellenbosch University	Cape Town, South Africa	Author	
Andrew	Redfern		MBChB, FCPPaed, M Phil	Department of Pediatrics and Child Health, Stellenbosch University	Cape Town, South Africa	Author	

Supplemental Online Content: Nonauthor Collaborators

*Indicates required information. Only first name, last name, and suffix will appear in PubMed.

*First Name and Middle Initial(s)	*Last Name	*Suffix (eg, Jr, III)	Academic Degrees	Institution	Location (city, state/province, country)	Role or Contribution, eg, chair, principal investigator	Group (if more than 1 Group listed in the byline) and/or Subgroup (eg, Steering Committee)
Angela	Dramowski		MBChB, PhD	Department of Pediatrics and Child Health, Stellenbosch University	Cape Town, South Africa	Author	
Natasha	O'Connell		MBChB, FCPPaed, MMed	Department of Pediatrics and Child Health, Stellenbosch University	Cape Town, South Africa	Author	
Michel T.	Pipo		MD	Department of Public Health, Centre Interdisciplinaire de Recherche en Ethnopharmacologie, Faculty of Medicine, Université Notre-Dame du Kasayi	Kananga, Democratic Republic of the Congo	Author	
Marc B.	Tshilanda		MD	Unit of Sickle Cell Disease and Clinical Research, Monkole Hospital Center	Kinshasa, Democratic Republic of the Congo	Author	
Liliane N.	Byamungu		MD, MSc	Department of Pediatrics and Child Health, School of Clinical Medicine, College of Health Sciences, University of KwaZulu Natal	Durban, South Africa	Author	

Supplemental Online Content: Nonauthor Collaborators

*Indicates required information. Only first name, last name, and suffix will appear in PubMed.

***First Name and Middle Initial(s)**	***Last Name**	***Suffix (eg, Jr, III)**	Academic Degrees	Institution	Location (city, state/province, country)	Role or Contribution, eg, chair, principal investigator	Group (if more than 1 Group listed in the byline) and/or Subgroup (eg, Steering Committee)
Refiloe	Masekela		MBChB, PhD	Department of Pediatrics and Child Health, School of Clinical Medicine, College of Health Sciences, University of KwaZulu Natal	Durban, South Africa	Author	
Prakash M.	Jeena		MBChB, PhD	Department of Pediatrics and Child Health, School of Clinical Medicine, College of Health Sciences, University of KwaZulu Natal	Durban, South Africa	Author	
Ashendri	Pillay		MBChB	Department of Pediatrics and Child Health, School of Clinical Medicine, College of Health Sciences, University of KwaZulu Natal	Durban, South Africa	Author	
Onesmus W.	Gachuno		MBChB, MMed, PGDRM	Department of Obstetrics and Gynecology, University of Nairobi	Nairobi, Kenya	Author	
John	Kinuthia		MBChB, MMed, MPH	Department of Research and Programs and Department of Reproductive Health, Kenyatta National Hospital	Nairobi, Kenya	Author	

*Indicates required information. Only first name, last name, and suffix will appear in PubMed.

***First Name and Middle Initial(s)**	***Last Name**	***Suffix (eg, Jr, III)**	Academic Degrees	Institution	Location (city, state/province, country)	Role or Contribution, eg, chair, principal investigator	Group (if more than 1 Group listed in the byline) and/or Subgroup (eg, Steering Committee)
Daniel K.	Ishoso		MD	Community Health Department, Kinshasa School of Public Health, University of Kinshasa	Kinshasa, Democratic Republic of the Congo	Author	
Emmanuela	Amoako		MBChB	Department of Pediatrics, Cape Coast Teaching Hospital	Cape Coast, Ghana	Author	
Elizabeth	Agyare		MBChB	Department of Microbiology, School of Medical Sciences, University of Cape Coast and Cape Coast Teaching Hospital	Cape Coast, Ghana	Author	
Evans K.	Agbeno		MBChB, MPH	Department of Obstetrics & Gynecology, School of Medical Sciences, University of Cape Coast and Cape Coast Teaching Hospital	Cape Coast, Ghana	Author	
Charles	Martyn-Dickens		MBChB	Pediatrics Infectious Diseases Unit, Komfo Anokye Teaching Hospital	Kumasi, Ghana	Author	
Justice	Sylverken		MD	Pediatrics Infectious Diseases Unit, Komfo Anokye Teaching Hospital	Kumasi, Ghana	Author	

*Indicates required information. Only first name, last name, and suffix will appear in PubMed.

*First Name and Middle Initial(s)	*Last Name	*Suffix (eg, Jr, III)	Academic Degrees	Institution	Location (city, state/province, country)	Role or Contribution, eg, chair, principal investigator	Group (if more than 1 Group listed in the byline) and/or Subgroup (eg, Steering Committee)
Anthony	Enimil		MBChB	Pediatrics Infectious Diseases Unit, Komfo Anokye Teaching Hospital	Kumasi, Ghana	Author	
Aishatu M.	Jibril		MBBS	Department of Pediatrics, College of Medical Sciences, Ahmadu Bello University	Zaria, Nigeria	Author	
Asara M.	Abdullahi		MBBS	Department of Internal Medicine, College of Medical Sciences, Ahmadu Bello University	Zaria, Nigeria	Author	
Oma	Amadi		MBBS	Department of Pediatrics, Asokoro District Hospital	Abuja, Nigeria	Author	
Umar M.	Umar		MD	Department of Internal Medicine, Asokoro District Hospital	Abuja, Nigeria	Author	
Lovemore N.	Sigwadhi		MSc	Division of Epidemiology and Biostatics, Department of Global Health, Stellenbosch University Faculty of Medicine and Health Sciences	Cape Town, South Africa	Author	
Michel P.	Hermans		MD, PhD	Department of Endocrinology and Nutrition, Cliniques Universitaires St-Luc	Brussels, Belgium	Author	

*Indicates required information. Only first name, last name, and suffix will appear in PubMed.

***First Name and Middle Initial(s)**	***Last Name**	***Suffix (eg, Jr, III)**	Academic Degrees	Institution	Location (city, state/province, country)	Role or Contribution, eg, chair, principal investigator	Group (if more than 1 Group listed in the byline) and/or Subgroup (eg, Steering Committee)
John O.	Otokoye		MD, MPH	World Health Organization, Health Emergencies Program, COVID-19 Response	Democratic Republic of the Congo	Author	
Placide	Mbala-Kingebeni		MD, PhD	National Institute of Biomedical Research (INRB) and Department of Medical Microbiology and Virology, Faculty of Medicine, University of Kinshasa	Kinshasa, Democratic Republic of the Congo	Author	
Alimuddin	Zumla		MD, PhD	Division of Infection and Immunity, Centre for Clinical Microbiology, University College London, and NIHR Biomedical Research Centre, UCL Hospitals NHS foundation Trust	London, UK	Author	
Hellen T.	Aanyu		MBChB	Department of Pediatrics, Mulago Hospital	Kampala, Uganda	Author	
Philippa	Musoke		MBChB, PhD	Department of Pediatrics and child health, College of Health Sciences, Makerere University	Kampala, Uganda	Author	

Supplemental Online Content: Nonauthor Collaborators

*Indicates required information. Only first name, last name, and suffix will appear in PubMed.

***First Name and Middle Initial(s)**	***Last Name**	***Suffix (eg, Jr, III)**	Academic Degrees	Institution	Location (city, state/province, country)	Role or Contribution, eg, chair, principal investigator	Group (if more than 1 Group listed in the byline) and/or Subgroup (eg, Steering Committee)
Fatima	Suleman		BPharm, PhD	Discipline of Pharmaceutical Sciences, University of KwaZulu Natal	Durban, South Africa	Author	
Emilia V.	Noormahomed		MD, PhD	Faculty of Medicine, Eduardo Mondlane University	Maputo, Mozambique	Author	
Mary G.	Fowler		MD	Department of Pathology, Johns Hopkins University School of Medicine	Baltimore, MD, USA	Author	
Leon	Tshilolo		MD, PhD	Department of Pediatrics, Official University of Mbuji-Mayi (UOM)	Kinshasa, Democratic Republic of the Congo	Author	
Mariana	Kruger		MMed, M Phil, PhD	Department of Pediatrics and Child Health, Stellenbosch University	Cape Town, South Africa	Author	
Gerald	Smith		PhD	Department of Real World & Advanced Analytics	Cytel, Vancouver, Canada	Author	
Philip J.	Rosenthal		MD	Department of Medicine, Division of Infectious Diseases, University of California	San Francisco, CA, USA	Author	
Edward J.	Mills		PhD	Department of Real World & Advanced Analytics	Cytel, Vancouver, Canada	Author	

*Indicates required information. Only first name, last name, and suffix will appear in PubMed.

***First Name and Middle Initial(s)**	***Last Name**	***Suffix (eg, Jr, III)**	Academic Degrees	Institution	Location (city, state/province, country)	Role or Contribution, eg, chair, principal investigator	Group (if more than 1 Group listed in the byline) and/or Subgroup (eg, Steering Committee)
Lawal W.	Umar		MBBS	Department of Pediatrics, College of Medical Sciences, Ahmadu Bello University	Zaria, Nigeria	Author	
John W.	Mellors		MD	Department of Medicine, Division of Infectious Diseases, University of Pittsburgh School of Medicine	Pittsburgh, PA, USA	Author	
Prisca	Adejumo		RN, PhD	Department of Nursing, University of Ibadan	Ibadan, Nigeria	Author	
Nelson K.	Sewankambo		MD	School of Medicine, College of Health Sciences, Makerere University	Kampala, Uganda	Author	
Mark J.	Siedner		MD	Harvard Medical School and Massachusetts General Hospital	Boston, MA, USA	Author	
Richard J.	Deckelbaum		MD	Columbia University Irving Medical Center	New York, NY, USA	Author	
Jean-Jacques	Muyembe-Tamfum		MD, PhD	National Institute of Biomedical Research (INRB) and Department of Medical Microbiology and Virology, Faculty of Medicine, University of Kinshasa	Kinshasa, Democratic Republic of the Congo	Author	

Supplemental Online Content: Nonauthor Collaborators

*Indicates required information. Only first name, last name, and suffix will appear in PubMed.

***First Name and Middle Initial(s)**	***Last Name**	***Suffix (eg, Jr, III)**	Academic Degrees	Institution	Location (city, state/province, country)	Role or Contribution, eg, chair, principal investigator	Group (if more than 1 Group listed in the byline) and/or Subgroup (eg, Steering Committee)
Lynne M.	Mofenson		MD	Elizabeth Glaser Pediatric AIDS Foundation	Washington DC, USA	Author	
Nancy	Mongweli		MBChB	Department of Research and Programs and Department of Reproductive Health, Kenyatta National Hospital	Nairobi, Kenya	Non-author collaborator	
Peter S.	Nyasulu		PhD, MScMed	Division of Epidemiology and Biostatics, Department of Global Health, Stellenbosch University Faculty of Medicine and Health Sciences	Cape Town, South Africa	Non-author collaborator	
Joule	Madinga		MD	World Health Organization, Health Emergencies Program, COVID-19 Response	Democratic Republic of the Congo	Non-author collaborator	
Christian Bongo-Pasi	Nswe		MD	Department of Public Health, Centre Interdisciplinaire de Recherche en Ethnopharmacologie, Faculty of Medicine, Université Notre-Dame du Kasayi	Kananga, Democratic Republic of the Congo	Non-author collaborator	

Supplemental Online Content: Nonauthor Collaborators

*Indicates required information. Only first name, last name, and suffix will appear in PubMed.

***First Name and Middle Initial(s)**	***Last Name**	***Suffix (eg, Jr, III)**	Academic Degrees	Institution	Location (city, state/province, country)	Role or Contribution, eg, chair, principal investigator	Group (if more than 1 Group listed in the byline) and/or Subgroup (eg, Steering Committee)
Jean-Marie N.	Kayembe		MD, PhD	Department of Medicine, Faculty of Medicine, University of Kinshasa	Kinshasa, Democratic Republic of the Congo	Non-author collaborator	
Abdon	Mukalay		MD, PhD, MPH	Faculty of Medicine and School of Public Health, University of Lubumbashi	Lubumbashi, Democratic Republic of the Congo	Non-author collaborator	
Alfred	Mteta		MD, PhD	Kilimanjaro Christian Medical University College	Moshi, United Republic of Tanzania	Non-author collaborator	
Aster	Tsegaye		MSc, PhD	Department of Medical Laboratory Sciences, College of Health Sciences, Addis Ababa University	Addis Ababa, Ethiopia	Non-author collaborator	
Don Jethro M.	Landu		MD, PhD, MPH	Department of Public Health, Centre Interdisciplinaire de Recherche en Ethnopharmacologie, Faculty of Medicine, Université Notre-Dame du Kasayi	Kananga, Democratic Republic of the Congo	Non-author collaborator	

Supplemental Online Content: Nonauthor Collaborators

*Indicates required information. Only first name, last name, and suffix will appear in PubMed.

***First Name and Middle Initial(s)**	***Last Name**	***Suffix (eg, Jr, III)**	Academic Degrees	Institution	Location (city, state/province, country)	Role or Contribution, eg, chair, principal investigator	Group (if more than 1 Group listed in the byline) and/or Subgroup (eg, Steering Committee)
Serge	Zigabe		MD	Hôpital Provincial Général de Référence de Bukavu, Département de Pédiatrie, Service de Néonatologie, Université Catholique de Bukavu	Bukavu, Democratic Republic of the Congo	Non-author collaborator	
Ameena	Goga		MBChB, PhD	South African Medical Research Council	Cape Town, South Africa	Non-author collaborator	
Rodney	Ehrlich		MBChB, PhD	University of Cape Town School of Public Health and Family Medicine	Cape Town, South Africa	Non-author collaborator	
Andre P.	Kengne		MD, PhD	South African Medical Research Council's Non-Communicable Diseases Research Unit	Cape Town, South Africa	Non-author collaborator	
John L.	Johnson		MD	Case Western Reserve University	Cleveland, OH, USA	Non-author collaborator	
Peter	Kilmarx		MD, MPH	US National Institutes of Health/Fogarty International Center	Bethesda, MD, USA	Non-author collaborator	
Birhanu	Ayele		PhD	Stellenbosch University	Cape Town, South Africa	Non-author collaborator	
Ireneous N.	Dasoberi			AFREhealth Executive Secretariat	Kumasi, Ghana	Administration	
Clara	Sam-Woode			AFREhealth Executive Secretariat	Kumasi, Ghana	Administration	
Georgina	Yeboah			AFREhealth Executive Secretariat	Kumasi, Ghana	Administration	

Supplemental Online Content: Nonauthor Collaborators

*Indicates required information. Only first name, last name, and suffix will appear in PubMed.

***First Name and Middle Initial(s)**	***Last Name**	***Suffix (eg, Jr, III)**	Academic Degrees	Institution	Location (city, state/province, country)	Role or Contribution, eg, chair, principal investigator	Group (if more than 1 Group listed in the byline) and/or Subgroup (eg, Steering Committee)
Chibueze	Adirieje		MPH	Central and West Africa Implementation Science Alliance (CAWISA) and the International Research Center of Excellence, Institute of Human Virology Nigeria	Abuja, Nigeria	Research Assistant	
